# Pitfalls of using video‐EEG for a trial endpoint in children aged <4 years with focal seizures

**DOI:** 10.1002/acn3.51999

**Published:** 2024-02-06

**Authors:** Ali Bozorg, Cynthia Beller, Lori Jensen, Alexis Arzimanoglou, Catherine Chiron, Dennis Dlugos, John Gaitanis, James W. Wheless, Carrie McClung

**Affiliations:** ^1^ UCB Pharma Morrisville North Carolina USA; ^2^ Department of Paediatric Clinical Epileptology, Sleep Disorders and Functional Neurology University Hospitals of Lyon (HCL), Member of the ERN EpiCARE Lyon France; ^3^ Epilepsy Unit San Juan de Dios Children's Hospital, Member of the ERN EpiCARE, Universitat de Barcelona Barcelona Spain; ^4^ Inserm U1141 and Necker‐Enfants Malades Hospital Paris France; ^5^ Departments of Neurology and Pediatrics Children's Hospital of Philadelphia, Perelman School of Medicine at the University of Pennsylvania Philadelphia Pennsylvania USA; ^6^ Tufts Medical Center Boston Massachusetts USA; ^7^ Le Bonheur Comprehensive Epilepsy Program & Neuroscience Institute, Le Bonheur Children's Hospital University of Tennessee Health Science Center Memphis Tennessee USA; ^8^ Present address: Otsuka Pharmaceutical Development & Commercialization, Inc. Rockville Maryland USA; ^9^ Present address: Brown Medical School Providence Rhode Island USA

## Abstract

**Objective:**

Double‐blind, randomized, and placebo‐controlled trial SP0967 (NCT02477839/2013‐000717‐20) did not demonstrate superior efficacy of lacosamide versus placebo in patients aged ≥1 month to <4 years with uncontrolled focal seizures, per ≤72 h video‐electroencephalogram (video‐EEG)‐based primary endpoints (reduction in average daily frequency of focal seizures at end‐of‐maintenance [EOM] versus end‐of‐baseline [EOB], patients with ≥50% response). This was unexpected because randomized controlled trial SP0969 (NCT01921205) showed efficacy of lacosamide in patients aged ≥4 to <17 years with uncontrolled focal seizures. SP0969's primary endpoint was based on seizure diary instead of video‐EEG, an issue with the latter being inter‐reader variability. We evaluated inter‐reader agreement in video‐EEG interpretation in SP0967, which to our knowledge, are the first such data for very young children with focal seizures from a placebo‐controlled trial.

**Methods:**

Local investigator and central reader agreement in video‐EEG interpretation was analyzed post hoc.

**Results:**

Analysis included 105 EOB and 98 EOM video‐EEGs. Local investigators and central reader showed poor agreement based on ≥2 focal seizures at EOB (Kappa = 0.01), and fair agreement based on ≥2 focal seizures at EOM (Kappa = 0.23). Local investigator and central reader seizure count interpretations varied substantially, particularly for focal seizures, but also primary generalized and unclassified epileptic seizures, at both timepoints.

**Interpretation:**

High inter‐reader variability and low inter‐reader reliability of the interpretation of seizure types and counts prevent confident conclusion regarding the lack of efficacy of lacosamide in this population. We recommend studies in very young children do not employ video‐EEGs exclusively for accurate study inclusion or as an efficacy measure.

## Introduction

Adequately powered, controlled, and prospective trials of anti‐seizure medications (ASMs) in young children are lacking, which has led to most children with early‐life seizures being prescribed ASMs off‐label, without adequate evidence of efficacy and safety.^1^ For the treatment of focal (partial‐onset) seizures, the most common seizure type in children and adults,^2^, ^3^ only five ASMs are approved in the United States (US) for patients aged <4 years (≥1 month of age: brivaracetam [BRV],^4^ lacosamide [LCM],^5^ levetiracetam [LEV],^6^, ^7^ and pregabalin^8^; ≥2 years of age: topiramate [TPM]^9^). In the European Union (EU), only four ASMs are approved for this age group (≥1 month of age: LEV^10^; ≥2 years of age: BRV,^11^ LCM,^12^ and TPM^13^). At the time of this trial (SP0967), only LEV and TPM were approved for this age group in the US and EU.

The primary objective of SP0967, a Phase III, double‐blind, randomized, placebo (PBO)‐controlled trial in 25 countries, was to evaluate the efficacy of LCM administered concomitantly with 1–3 ASMs in patients aged ≥1 month to <4 years with uncontrolled focal seizures. As assessed by reduction in average daily frequency (ADF) of focal seizures and the proportion of patients with a ≥50% response based on ≤72 h video‐electroencephalogram (video‐EEG) at the end of the maintenance period (EOM) versus the end of the baseline period (EOB), SP0967 did not demonstrate superior efficacy of LCM versus PBO.^14^ This was unexpected because LCM showed efficacy in patients with uncontrolled focal seizures in the adjacent age group of ≥4 to <17 years, in another double‐blind, randomized, PBO‐controlled trial (SP0969; NCT01921205).^15^ SP0969 assessed efficacy using seizure diaries instead of video‐EEGs, and had much longer treatment and evaluation periods than SP0967 (6‐week titration, 10‐week maintenance).

As SP0967 enrolled young children/infants with the same seizure type, there was no mechanistic reason to suspect lack of efficacy. Both trials recruited patients with uncontrolled focal seizures on a stable regimen of 1–3 ASMs and used LCM doses that achieved a therapeutic serum range (previously established in adults and older children).^5^, ^12^ This similarity suggests the difference in efficacy is not solely because of age differences and may relate to how seizures were counted (primary endpoints based on video‐EEG in SP0967 vs. seizure diary data in SP0969).

As discussed in Auvin et al.,^1^ the traditional methodology of PBO‐controlled trials for focal seizures in children and infants aged <4 years, which is based on video‐EEG, is impractical and unreliable. Besides ethical concerns of these video‐EEG‐based designs (requirement of two hospitalizations of 48–72 h, exclusion of patients with low seizure burden and exposure to PBO for patients who could benefit from off‐label access to the active drug), the feasibility of video‐EEGs as a measure of the primary endpoint has been questioned.^1^ In particular, the level of inter‐reader agreement in EEG/video‐EEG interpretation has long been an issue in children and adults.^16^, ^17^ We report data on inter‐reader agreement in video‐EEG interpretation from SP0967, which to our knowledge, are the first such data for very young children with focal seizures from a PBO‐controlled trial.

## Methods

### Trial design

**Figure 1:**
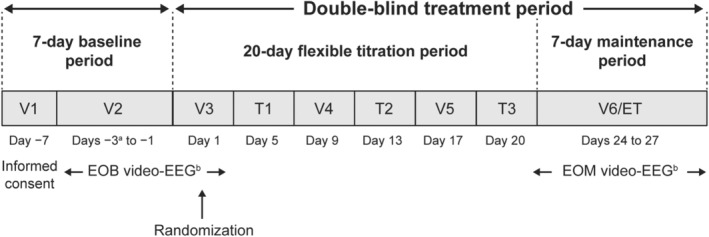
SP0967 trial design. EOB, end‐of‐baseline period; EOM, end‐of‐maintenance period; ET, early termination; T, telephone contact; V, visit. ^a^A maximum of 7 days could be added to the period between V1 and V2 in the event that additional time was needed to access inpatient facilities to perform the video‐EEG. ^b^The video‐EEG (up to 72 h of continuous recording, with every attempt to obtain ≥48 h of interpretable recording) was conducted in an inpatient setting. Upon completion of the EOB video‐EEG (V2 and V3), the investigator assessed the electrographic seizure count and confirmed the selection criteria were met. If the patient met all selection criteria, including the requisite number of seizures based on the video‐EEG data, the patient was randomized at V3. Patients who discontinued on or before day 20 did not require an EOM video‐EEG.

SP0967 (ClinicalTrials.gov NCT02477839, EudraCT 2013‐000717‐20) was a Phase III, multicenter, double‐blind, randomized, PBO‐controlled, parallel‐group trial to evaluate the efficacy and safety of LCM as adjunctive treatment in patients ≥1 month to <4 years of age with uncontrolled focal seizures. The methodology and primary results are described elsewere.^14^ In brief, SP0967 comprised a 7‐day baseline period and a 20‐day blinded titration period (with trial medication dosing flexibility allowed based on tolerability) to attain the target dose of trial medication for the 7‐day blinded maintenance period (LCM 8–12 mg/kg/day or matching PBO; no adjustments to trial medication dose were allowed) (Fig. 1).

SP0967 was conducted in accordance with applicable regulatory and International Council for Harmonisation‐Good Clinical Practice requirements, the Declaration of Helsinki, and local laws. The protocol and amendments were reviewed by a national, regional, or independent ethics committee or institutional review board. Written informed consent was provided by parents or legal representatives of all patients.

### Use of video‐EEG in SP0967

SP0967 enrolled patients with uncontrolled epilepsy and frequent seizures in order to limit trial duration, and utilized video‐EEG to optimize the detection of seizures, which can be subtle during infancy. To be eligible for enrollment, patients were required to have ≥2 focal seizures with or without secondary generalization during the EOB video‐EEG, which had a duration of up to 72 h. Electrographic seizures were defined as recognizable ictal patterns on an EEG involving ≥2 contiguous electrodes. The seizures were to have been initiated as a unilateral or strongly asymmetric abnormal epileptiform discharge lasting a total of >10 seconds.^18^

Region‐specific primary endpoints of the trial were also based on video‐EEG, following the design of a previous LEV trial in the same age group of ≥1 month to <4 years (N01009; NCT00175890), which demonstrated the efficacy of LEV for the treatment of focal seizures in this age group.^18^ According to requirements from the US Food and Drug Administration (FDA) and the European Medicines Agency (EMA), in trial SP0967 2 region‐specific primary efficacy variables were defined based on electrographic focal seizures with or without clinical correlate, depending on patient age. The request for electrographic seizures may be valid for nonmotor seizures, particularly in young children, but this is not the type of seizure usually evaluated in trials. In line with regulatory agency feedback, for infants ≥1 month to ≤6 months of age, focal seizure frequency was based on electrographic seizures (with or without a clinical correlate); for children >6 months to <4 years of age, focal seizure frequency was based on electrographic seizures with an accompanying clinical correlate only.

The region‐specific primary efficacy variables were the change in ADF of electrographic focal seizures as measured on the EOM video‐EEG compared with the EOB video‐EEG (US) and the proportion of patients experiencing a ≥50% reduction in their ADF of electrographic focal seizures during the maintenance period (EU). The video‐EEGs were obtained using up to 72 h of continuous recording, with every attempt to obtain ≥48 h of interpretable recording.

Per the original protocol, local investigators were responsible for reviewing EOB video‐EEGs to determine patient eligibility with respect to focal seizure frequency, and a central video‐EEG reader reviewed all video‐EEGs and established the seizure counts used in primary and secondary efficacy analyses at a later point in time. It was not operationally feasible to have a central reader assess patient eligibility, given that the selected sites were located in multiple countries across different time zones, and eligibility assessments were conducted across all days of the week.

During the course of the trial, it emerged that there was high variability in seizure counts between the investigators and the central reader. To address this, a protocol amendment was implemented after discussion with regulatory agencies, to remove the central reader and extend the investigators' responsibility from assessing seizure counts at EOB for eligibility only, to assessing seizure counts at both EOB and EOM for efficacy analyses as well, including the patients who had already completed the trial.

### Analyses of inter‐reader variability in video‐EEG interpretation

**Figure 2:**
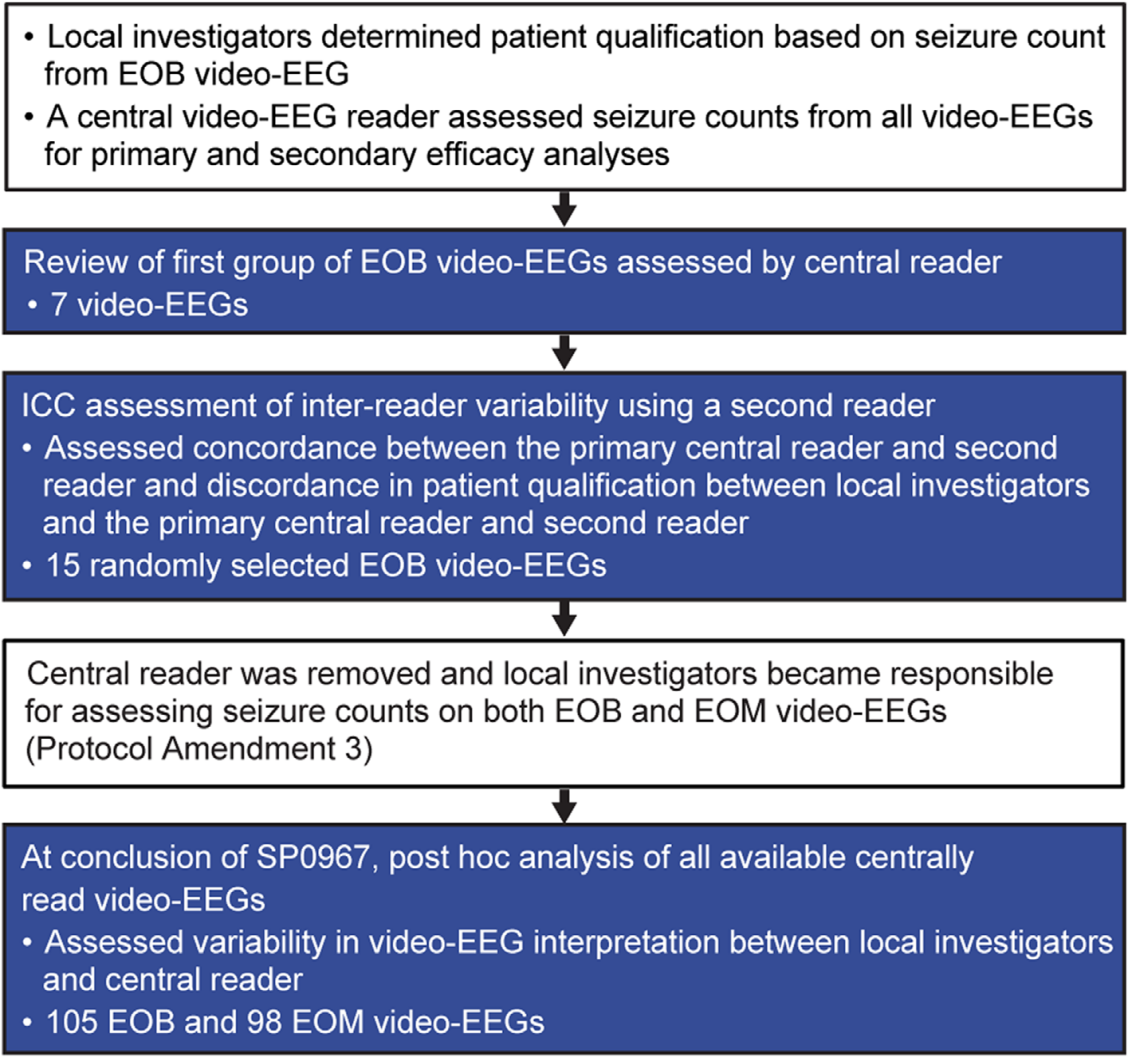
Flow chart summarizing analyses of inter‐reader variability in video‐EEG interpretation and video‐EEG‐related protocol amendments. EOB, end‐of‐baseline period; EOM, end‐of‐maintenance period; ICC, intra‐class correlation.

Analyses of inter‐reader variability in video‐EEG interpretation in SP0967 are summarized in Figure 2. The first EOB video‐EEGs read by the primary central reader (the designated central reader in the trial) for the efficacy analyses were reviewed. A formal assessment of inter‐reader variation was then carried out using a second reader. This intra‐class correlation (ICC) assessment of 15 randomly selected EOB video‐EEG readings evaluated the concordance between the two readers and, secondarily, the discordance in patient qualification between the investigators and the primary central reader and the second reader.

Upon conclusion of the trial, a post hoc analysis of all available centrally read video‐EEGs was conducted to assess variability in video‐EEG interpretation between local investigators and the central reader. This analysis evaluated Cohen's Kappa statistic^19^ relative to the following cut points: poor agreement (<0.20), fair agreement (≥0.20 to ≤0.40), moderate agreement (>0.40 to ≤0.60), good agreement (>0.60 to ≤0.80), and very good agreement (>0.80 to 1.00).^20^

## Results

### Review of first group of EOB video‐EEGs assessed by the central reader

A review of the first seven EOB video‐EEGs read by the central reader (for the efficacy analyses), which the local investigator had deemed as meeting the eligibility threshold of ≥2 focal seizures, showed that as per central reader assessment, only 28.6% (2 out of 7) of patients met the threshold. In contrast, the protocol anticipated that there would be no difference between the local investigator and the central reader in interpretation of the EOB video‐EEG for 95% of enrolled patients (based on the LEV N01009 pediatric trial^18^).

### Assessment of inter‐reader variability using a second reader

To establish whether the high variability could be attributed to the primary central reader, a formal assessment of inter‐reader variation was carried out using a second reader. The results showed only moderate agreement between the primary central reader and the second reader on the assessment of focal seizure counts. The ICC for log‐transformed focal seizure counts for the two readers was 0.632, which falls into the moderate agreement or gray zone category based on Landis and Koch agreement measures for categorical data.^21^

There was also a high degree of discordance between the investigators and the primary central reader and second reader in the proportion of patients who qualified for enrollment into the trial. Of the 15 cases reviewed, who were screened between March 2016 and February 2017 and had all been assessed by the investigators as having ≥2 focal seizures, only 6 (40.0%) (95% confidence interval [CI] = 16.3–67.7) and 5 (33.3%) (95% CI = 11.8–61.6) qualified according to the assessment by the primary central reader and second reader, respectively. The primary central reader and second reader agreed on ≥2 focal seizures in only 4 (26.7%) cases.

### Post hoc analysis of variability between investigators and central reader

**Figure 3:**
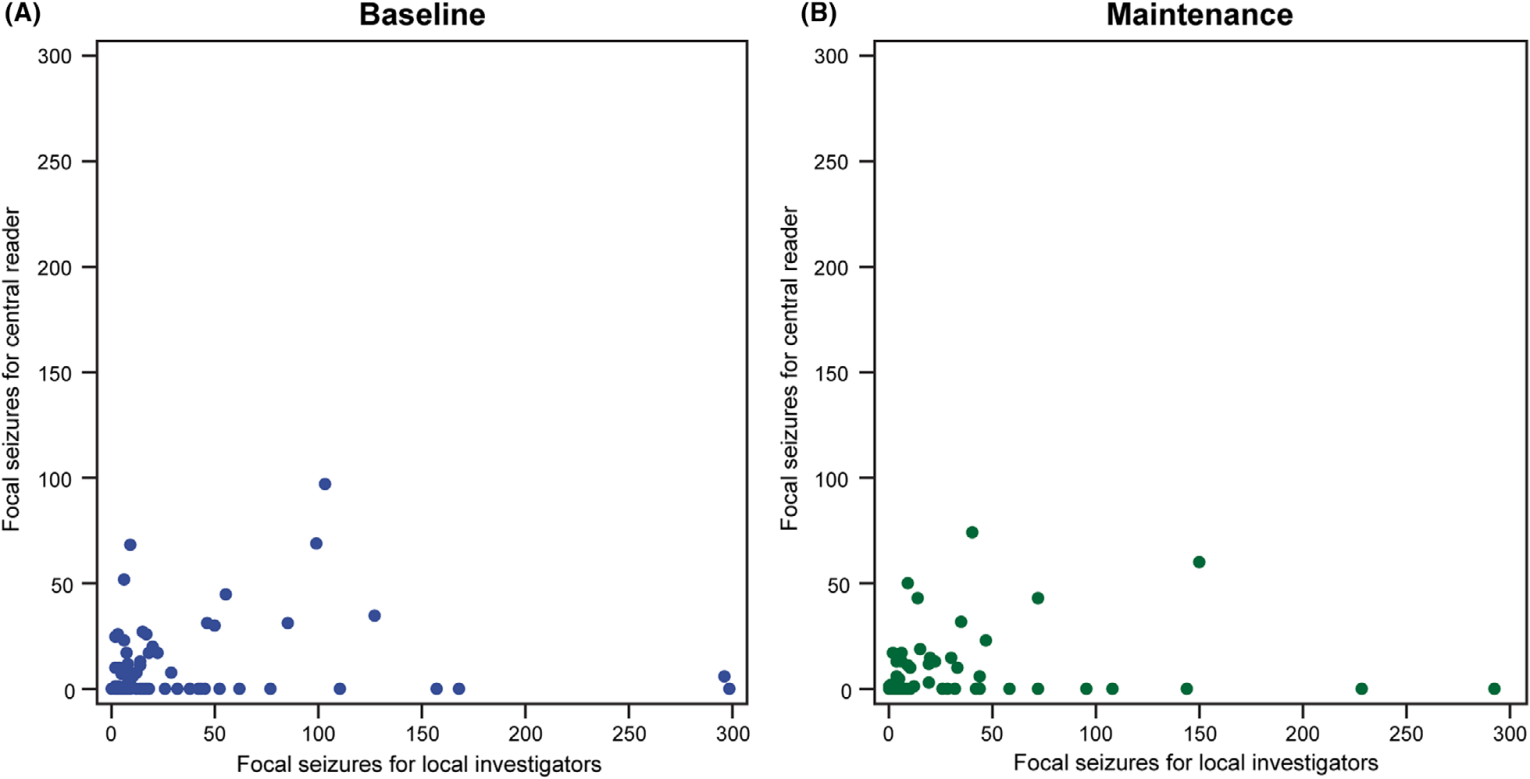
Number of focal seizures for each patient, as assessed by local investigators versus central reader, by trial period (full analysis set—patients with central reader and local investigators data per trial period). (A) Baseline, and (B) maintenance. The data for one patient are not displayed as the values are outliers.

**Figure 4:**
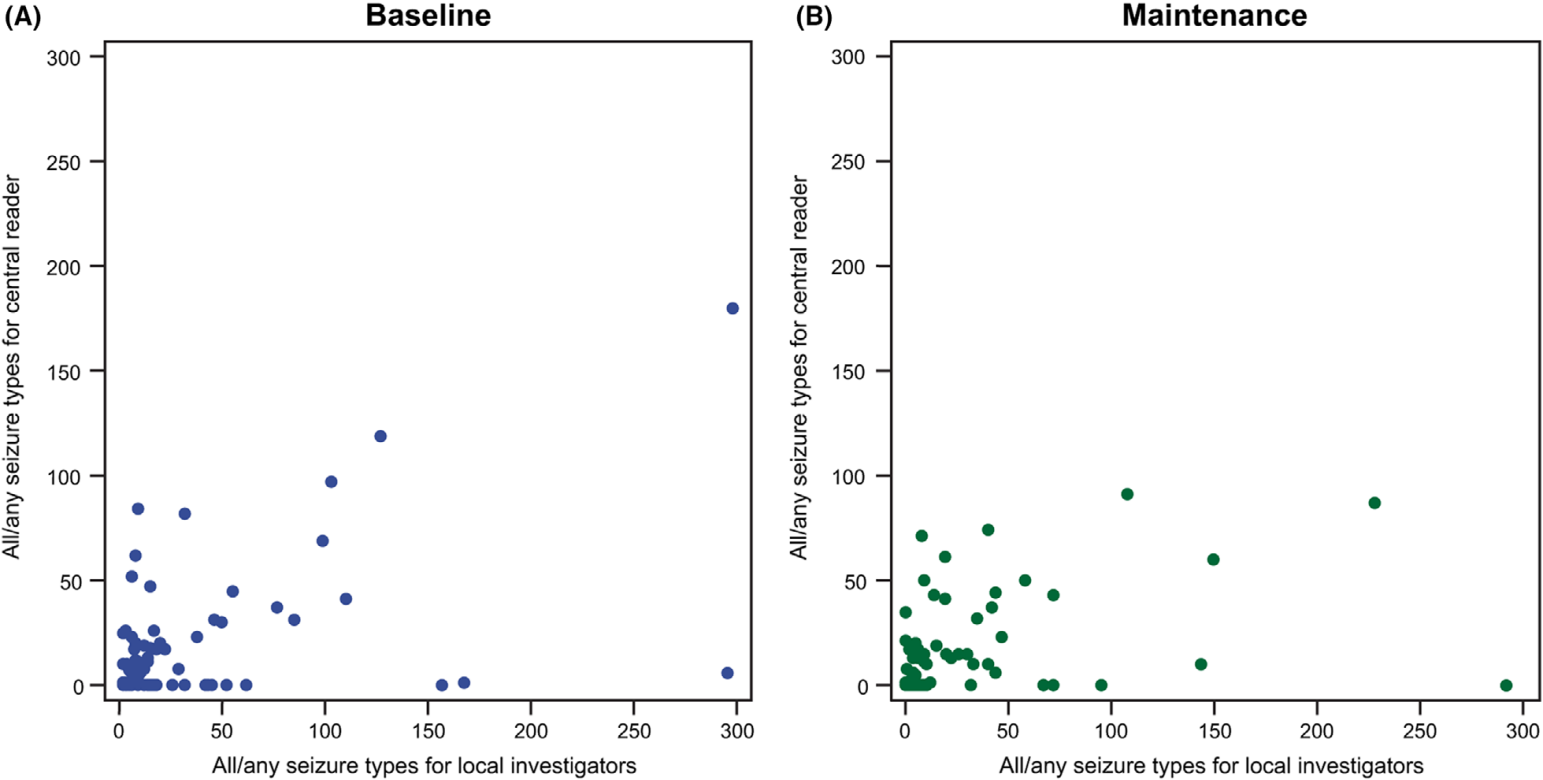
Number of seizures (all/any type) for each patient, as assessed by local investigators versus central reader, by trial period (full analysis set—patients with central reader and local investigators data per trial period). (A) Baseline, and (B) maintenance. The data for two patients are not displayed as the values are outliers.

The post hoc analysis of all available centrally read video‐EEGs included a total of 105 EOB video‐EEGs and 98 EOM video‐EEGs. As measured by Kappa statistics for patient qualification based on the presence of ≥2 focal seizures, local investigators and the central reader were categorized as in poor agreement at EOB (Table 1) and as in fair agreement at EOM (Table 2). At EOB, a difference of 64.7% in reader interpretation was observed: the proportion of patients with ≥2 focal seizures was 99.0% per local investigator assessment and 34.3% per central reader assessment (Table 1). The number of focal seizures for local investigators versus the central reader at EOB and EOM showed no obvious linear relationship (Fig. 3). The number of all/any type of seizure for local investigators versus the central reader at EOB and EOM also showed no obvious linear relationship (Fig. 4).

**Table 1:** Cross tabulation of focal seizure counts by local investigators versus central reader for the end of baseline period (full analysis set—patients with central reader and local investigators data for the end of baseline period).

Central reader	Local investigators		Row total *n* (%)	Kappa (95% CI)
	<2 focal seizures *n* (%)	≥2 focal seizures *n* (%)		
<2 focal seizures	1 (1.0)	68 (64.8)	69 (65.7)	0.01 (−0.01 to 0.03)
≥2 focal seizures	0	36 (34.3)	36 (34.3)	
Column total	1 (1.0)	104 (99.0)	105 (100)	

The Kappa statistic is provided between local investigators and the central reader regarding patient qualification based on ≥2 focal seizures.

CI, confidence interval.

**Table 2:** Cross tabulation of focal seizure counts by local investigators versus central reader for the end of maintenance period (full analysis set—patients with central reader and local investigators data for the end of maintenance period).

Central reader	Local investigators		Row total *n* (%)	Kappa (95% CI)
	<2 focal seizures *n* (%)	≥2 focal seizures *n* (%)		
<2 focal seizures	26 (26.5)	44 (44.9)	70 (71.4)	0.23 (0.12 to 0.34)
≥2 focal seizures	1 (1.0)	27 (27.6)	28 (28.6)	
Column total	27 (27.6)	71 (72.4)	98 (100)	

The Kappa statistic is provided between local investigators and the central reader regarding patient qualification based on ≥2 focal seizures.

CI, confidence interval.

**Figure 5:**
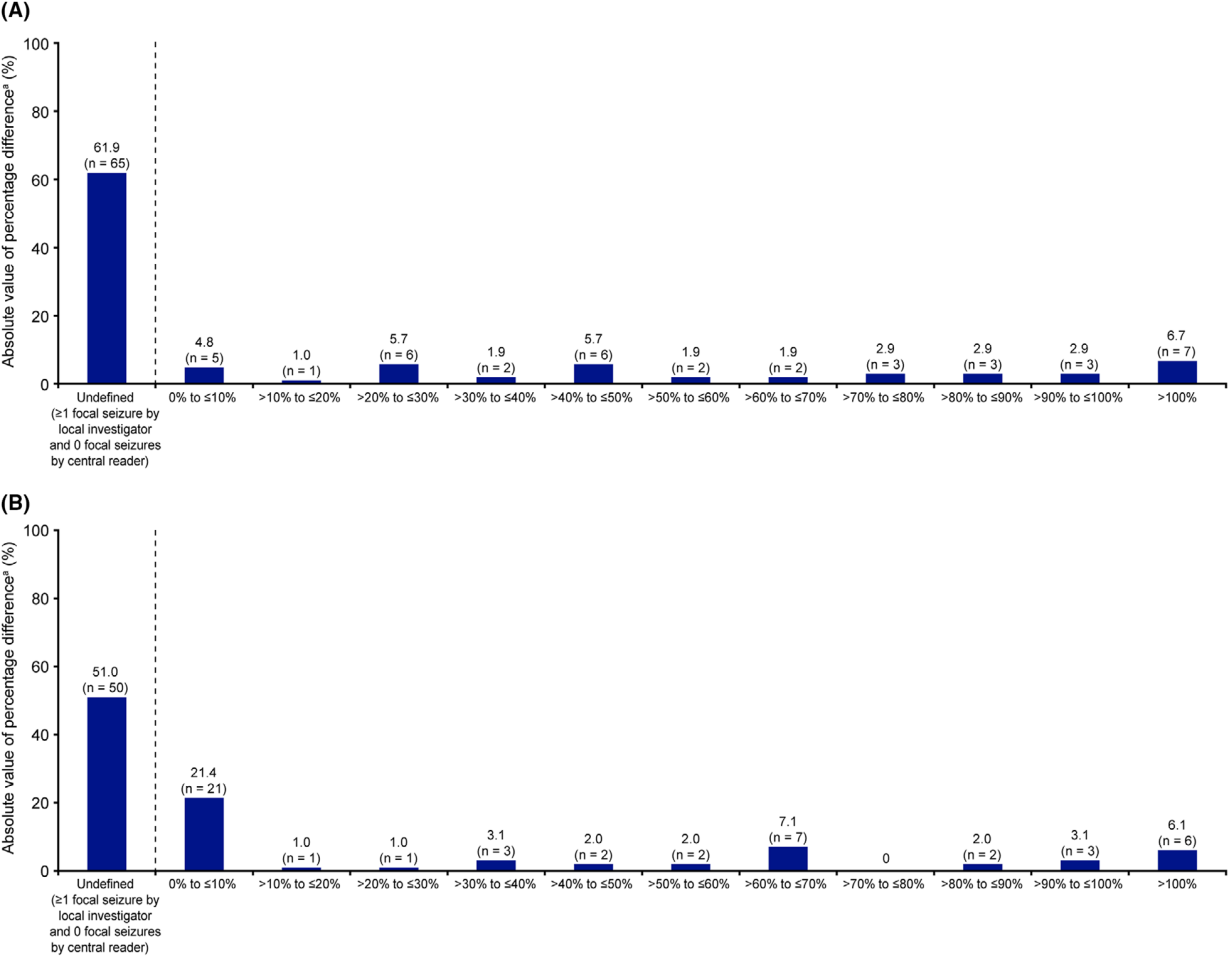
Percentage difference in focal seizure counts between local investigators and central reader, by trial period (full analysis set—patients with central reader and local investigators data per trial period). (A) Baseline (*N* = 105), and (B) maintenance (*N* = 98). ^a^Percentage difference was calculated as 100 × (local investigator − central reader)/central reader, and categorized based on the absolute value.

In the majority of cases at both EOB (61.9%) and EOM (51.0%), the central reader did not observe any focal seizures despite local investigator determination of presence of ≥1 focal seizure (Fig. 5). There was minimal discrepancy (≤10%) between local investigator and central reader interpretations of focal seizure count in only 4.8% of cases at EOB and 21.4% of cases at EOM. Overall, there was substantial variability between local investigator and central reader interpretations of seizure counts, particularly in the number of focal seizures, but also in the number of primary generalized and unclassified epileptic seizures, at both EOB (Table 3) and EOM (Table 4). However, there was minimal difference (median percentage difference: 0.00) in the number of interpretable hours of video‐EEG between local investigators and the central reader, at both EOB and EOM (Table 5).

**Table 3:** Seizure types as assessed by local investigators versus central reader, for the end of baseline period (*N* = 105; full analysis set—patients with central reader and local investigators data for the end of baseline period).

Central reader	Local investigators					
	No seizures of any type *n* (%)	Only Type 1 seizures *n* (%)	Only Type 2 seizures *n* (%)	Only Type 3 seizures *n* (%)	Only Type 1 and 2 seizures, only type 1 and 3 seizures, only type 2 and 3 seizures, or type 1 and 2 and 3 seizures *n* (%)	No Type 1 seizures *n* (%)
No seizures of any type	0^a^	49 (46.7)	0	1 (1.0)	0	1 (1.0)
Only Type 1 seizures	0	36 (34.3)^a^	0	0	0	0
Only Type 2 seizures	0	14 (13.3)	0^a^	0	0	0
Only Type 3 seizures	0	2 (1.9)	0	0^a^	0	0
Only Type 1 and 2 seizures, only Type 1 and 3 seizures, only Type 2 and 3 seizures, or Type 1 and 2 and 3 seizures	0	3 (2.9)	0	0	0^a^	0
No Type 1 seizures	0	65 (61.9)	0	1 (1.0)	0	0^a^

Type 1 seizures are focal (partial‐onset) seizures; Type 2 seizures are primary generalized seizures; Type 3 seizures are unclassified epileptic seizures.

^a^ Agreement between local investigators and the central reader for seizure type.

**Table 4:** Seizure types as assessed by local investigators versus central reader, for the end of maintenance period (*N* = 98; full analysis set—patients with central reader and local investigators data for the end of maintenance period).

Central reader	Local investigators					
	No seizures of any type *n* (%)	Only Type 1 seizures *n* (%)	Only Type 2 seizures *n* (%)	Only Type 3 seizures *n* (%)	Only Type 1 and 2 seizures, only type 1 and 3 seizures, only Type 2 and 3 seizures, or Type 1 and 2 and 3 seizures *n* (%)	No Type 1 seizures *n* (%)
No seizures of any type	13 (13.3)^a^	41 (41.8)	1 (1.0)	0	0	14 (14.3)
Only Type 1 seizures	1 (1.0)	25 (25.5)^a^	0	1 (1.0)	0	2 (2.0)
Only Type 2 seizures	2 (2.0)	8 (8.2)	0^a^	0	1 (1.0)	2 (2.0)
Only Type 3 seizures	1 (1.0)	0	0	0^a^	0	1 (1.0)
Only Type 1 and 2 seizures, only Type 1 and 3 seizures, only Type 2 and 3 seizures, or Type 1 and 2 and 3 seizures	0	4 (4.1)	0	0	0^a^	0
No Type 1 seizures	16 (16.3)	49 (50.0)	1 (1.0)	0	1 (1.0)	0^a^

Type 1 seizures are focal (partial‐onset) seizures; Type 2 seizures are primary generalized seizures; Type 3 seizures are unclassified epileptic seizures.

^a^ Agreement between local investigators and the central reader for seizure type.

**Table 5:** Absolute and percentage difference in interpretable hours of video‐EEG between local investigators and central reader, by trial period (full analysis set).

Trial period Statistic	Difference in interpretable hours (local investigator − central reader)	Percentage difference in interpretable hours^a^
*Baseline*		
*n*	105	104
Median	0.00	0.00
Min, max	−72.33, 71.27	−50.09, 2361.38
*Maintenance*		
*n*	98	98
Median	0.00	0.00
Min, max	−20.45, 31.77	−28.60, 80.94

Max, maximum; min, minimum.

^a^ Percentage difference was calculated as 100 × (local investigator − central reader)/central reader.

## Discussion

Our analyses showed substantial variability and low reliability between video‐EEG readers in their assessments of seizure counts and classification in young children/infants. Although differences in the ability of trained neurologists to agree on EEG/video‐EEG interpretation have been identified,^16^, ^17^ to our knowledge, this is the first assessment of inter‐reader reliability in a PBO‐controlled trial in children with focal seizures aged ≥1 month to <4 years, most of whom had difficult‐to‐treat seizures, and half of whom received PBO.^14^

Post hoc analysis of all available centrally read video‐EEGs showed poor agreement between local investigators and the central reader for focal seizure counts at EOB and fair agreement at EOM (Kappa statistics). In most cases at both timepoints, local investigators determined ≥1 focal seizure whereas the central reader determined none. Variability in focal seizure counts was not driven by seizure classification: for both focal seizures and seizures of all/any type, local investigator and central reader counts did not show a linear relationship. Local investigator and central reader seizure counts varied substantially, especially for focal seizures, but also primary generalized and unclassified epileptic seizures, at both timepoints. It appears that video‐EEG data quality was not an issue and the data were not the source of the variability in interpretation, as there was minimal difference in the number of interpretable hours of video‐EEG between local investigators and the central reader.

This raises a question on the reliability of video‐EEG as an efficacy measure, confirming published concerns about the utility of video‐EEG‐based endpoints in ASM trials in young children/infants.^1^ Video‐EEG is less reliable in younger children versus older children and adults because of difficulty determining whether the seizures are clearly focal.^17^ In the authors' experience, many young children, especially those aged <2 years, have unclear epilepsy syndromes with potentially multiple seizure types. Seizures in young children are more variable and may not have a clear onset and end, making a “seizure count” difficult.

Whether a signal is counted as a seizure or artifact depends on the clinical pattern/semiology, type of electric activity, alignment with the video track, and previous observation of similar seizures in the same child. Furthermore, there may be inconsistency in whether seizures that are captured on EEG but not on camera are counted as clinical seizures. For seizure detection on EEG, inter‐reader agreement is associated with the duration and rarity of seizures,^22^ their spatial extent, and whether the seizures are clinical or subclinical. Therefore, it is difficult for different readers to achieve consensus when interpreting video‐EEGs in young children,^17^ and occasionally readers disagree with their own earlier interpretation. For SP0967, the inclusion of 25 countries, with potentially different approaches to video‐EEG interpretation, may also have increased the variability. Although intra‐reader variability was not assessed because of lack of data, a study on EEG interpretation in critically ill adults showed that intra‐reader agreement was only modestly better than inter‐reader agreement.^16^

Video‐EEG‐based efficacy endpoints may not have been the only factor in the negative outcome of SP0967. Other trial limitations were the inclusion of sites across multiple regions where video‐EEG training methods may vary, inadequate site training in identifying seizures from video‐EEG, insufficiently clear rules and definitions for electrographic seizures, and an adjudication process that did not require central reader confirmation of the presence of ≥2 focal seizures before randomization. Addressing these issues may have improved the reliability of video‐EEG seizure counts.

### Comparison with other ASM trials in young children with focal seizures

The design of SP0967 was based on that of a double‐blind, randomized, PBO‐controlled, parallel‐group trial which had demonstrated the efficacy of adjunctive LEV in patients with focal seizures in the same age group (N01009).^18^ Patients in SP0967 had more drug‐resistant epilepsy than those in N01009. This may be related to the greater number of permitted concomitant ASMs (1–3 not including vagus nerve stimulation [VNS] vs. 1–2 including VNS), and the availability of additional ASMs (used both on‐label and off‐label) in the more than 10 years since the conduct of N01009. In SP0967, 27.6% and 27.3% of PBO and LCM patients, respectively, were taking >2 ASMs on the day of the first dose of trial medication, whereas in N01009 only 8.9% and 6.7% of PBO and LEV patients, respectively, had >2 concomitant ASMs at baseline.^18^

A0081042 was another double‐blind, randomized, PBO‐controlled, parallel‐group trial in patients with focal seizures aged 1 month to <4 years that employed video‐EEG.^23^ In the trial, adjunctive pregabalin 14 mg/kg/day significantly reduced focal seizure frequency versus PBO, although the lower dose (7 mg/kg/day) did not. Compared with SP0967, fewer patients in A0081042 were receiving >2 concomitant ASMs (range 12%–20% across treatment groups at randomization).^23^ The fixed‐dose treatment period was also slightly longer versus SP0967 (9 vs. 7 days).^14^, ^23^ In A0081042, investigators reviewed baseline video‐EEGs to determine patient eligibility per the required number of focal seizures, and a central reader reviewed video‐EEGs to determine focal seizure counts for efficacy assessments.^23^ To our knowledge, inter‐reader correlation in the trial has not been published.

A double‐blind, randomized, PBO‐controlled, parallel‐group trial evaluated adjunctive TPM in infants aged 1–24 months with focal seizures.^24^ The trial failed to demonstrate the superiority of TPM over PBO in its primary endpoint (percentage reduction in daily focal seizure rate from baseline to final assessment as recorded on 48‐h video‐EEG) at all doses tested (5, 15, and 25 mg/kg/day). Patients had 1–2 concomitant ASMs at baseline; those with functioning VNS were excluded. The double‐blind treatment period, including titration, was 20 days. The duration of the fixed‐dose portion was not specified, but patients were only exposed to the higher doses for a few days. The PBO effect was 13.1% for median percentage reduction from baseline in daily focal seizure rate (TPM 25 mg/kg/day: 20.4%) and 36% for 50% responder rate (TPM 25 mg/kg/day: 44%), which is higher than that in N01009 but lower than or similar to that in SP0967.^14^, ^18^, ^24^

### Use of video‐EEG in trials in young children with focal seizures and alternatives

Video‐EEG interpretation in young children can be challenging and the need for interpretation makes these assessments only semi‐objective. Our analysis shows that two qualified readers may provide different interpretations and seizure counts from the treating physician in young children with focal seizures. For clinical trials in this patient group, video‐EEG may have more value as a qualitative measure to determine eligibility than as an objective, quantitative measure of efficacy and the basis of the primary endpoint.

Other issues in PBO‐controlled trials with video‐EEG‐based endpoints include exposure to PBO when the active drug can be accessed off‐label, exclusion of patients with infrequent seizures, lack of generalizability to clinical practice, and the need for two hospitalizations of 48–72 h.^1^ Current video‐EEG‐based trial designs are also very complex and difficult to apply in real life. Therefore, efficacy measures not based on video‐EEG may be preferable. Examples include the longest time interval between seizures and seizure counts by caregivers. The latter is part of a novel trial design for children with focal seizures aged ≥1 month to <4 years proposed by the International League Against Epilepsy, in collaboration with the Pediatric Epilepsy Research Consortium,^1^ which came about partly because of the findings from SP0967. The time‐to‐event design involves seizure counting by caregivers based on previous video‐EEG/video validation of specific seizure semiologies. The duration of baseline and exposure to PBO or ineffective treatment are adjusted to the patient's seizure burden and response. This new design has not been validated in drug development; however, there is evidence to support its utility from a post hoc analysis of randomized, PBO‐controlled trials in patients with focal seizures aged 4–16 years.^25^

Another alternative to video‐EEG‐based PBO‐controlled trials is pharmacologic extrapolation from adults to children. Both LCM and BRV were recently approved by the FDA and EMA for the treatment of focal seizures in children based on extrapolation.^4^, ^5^, ^11^, ^12^, ^26^

## Conclusion

SP0967, a double‐blind, randomized, PBO‐controlled trial designed and conducted to provide efficacy and safety data in the very challenging population of patients aged ≥1 month to <4 years with uncontrolled focal seizures, did not demonstrate superior efficacy of adjunctive LCM versus PBO as measured by the primary endpoints (reduction in ADF of focal seizures and proportion of patients with ≥50% response) based on video‐EEG.^14^ However, high inter‐reader variability and low inter‐reader reliability of the interpretation of seizure types and counts based on video‐EEG, prevent confident conclusion regarding the lack of efficacy of LCM in this population. Based on our findings, we recommend future studies in very young children, especially where the children may be exposed to PBO, do not employ video‐EEG measures as an exclusive tool for accurate study inclusion or as a measure of efficacy. To improve the accuracy of seizure counting, we recommend pre‐identifying the clinical signs for each study participant from video‐EEGs or simple videos (including home videos), as proposed in a novel trial design.^1^

## Author Contributions

A.B., L.J., and C.M. contributed to trial concept or design, analysis or interpretation of data, and drafting/revising the manuscript for content. C.B. contributed to trial concept or design, analysis or interpretation of data, statistical analyses, and drafting/revising the manuscript for content. A.A., C.C., D.D., J.G., and J.W.W. contributed to acquisition of data, analysis or interpretation of data, and drafting/revising the manuscript for content.

## Conflicts of Interest

A. Bozorg was an employee and shareholder of UCB Pharma at the time of conduct of the trial. C. Beller, L. Jensen, and C. McClung are employees of UCB Pharma, and have received UCB Pharma stocks or stock options from their employment. A. Arzimanoglou has received consulting fees from Eisai, Jazz, Orion, Sanofi, and UCB Pharma; received payments for lectures, presentations, speaker bureaus, manuscript writing, or educational events from Eisai, Jazz, Orion, and UCB Pharma; participated on a Data Safety Monitoring Board or Advisory Board for UCB Pharma; taken a leadership or fiduciary role for the International League Against Epilepsy; and served as Coordinator for the European Reference Network EpiCARE. C. Chiron has received consulting fees from Bial; and received payments for lectures, presentations, speaker bureaus, manuscript writing, or educational events, and support for attending meetings and/or travel from Advicenne, Biocodex, Eisai, GW Pharma, Neuren, and Orphelia. D. Dlugos has nothing to report. J. Gaitanis has received speaking fee from Neurelis and payment for expert testimony from CRICO insurance. J.W. Wheless has received grants from Biohaven, Envision, Epiwatch, Longboard, Marinus, National Institutes of Health, Neurelis, Neuro Event Labs, Shainberg Foundation, SKLSI, Stoke, TSC Alliance, UCB Pharma, and Xenon; participated in speaker bureaus for Azurity, BioMarin, Eisai, Jazz, LivaNova, Neurelis, SKLSI, Supernus, and UCB Pharma; and served as a consultant for Azurity, BioMarin, Jazz, Marinus, and Neurelis.

## Data Availability

Underlying data from this manuscript may be requested by qualified researchers 6 months after product or indication approval in the United States and/or Europe, or global development is discontinued, and 18 months after trial completion. Investigators may request access to anonymized individual patient‐level data and redacted trial documents which may include: analysis‐ready datasets, study protocol, annotated case report form, statistical analysis plan, dataset specifications, and clinical study report. Before use of the data, proposals need to be approved by an independent review panel at www.Vivli.org and a signed data sharing agreement will need to be executed. All documents are available in English only, for a prespecified time, typically 12 months, on a password protected portal.
